# Acute Articular Rheumatism

**Published:** 1907-08

**Authors:** 


### ACUTE ARTICULAR RHEUMATISM.

Phillips gives the following treatment for acute articular rheumatism:

The patient, clothed in a flannel night-gown, should be kept at rest in a comfortable bed and should lie preferably between blankets. Abundant drinking of water is necessary to favor elimination and to make up for the fluid lost through free perspiration. Instead of plain water, cream of tartar water may be substituted. The bowels should be opened freely by the adminstration of calomel followed by a saline, and afterward kept moving regularly by cascara. Where the perspiration is free, the patient can be made comportable by sponging his body with water to which sodium bicarbonate has been added. The diet at first should consist of milk, although if the latter is not well borne there is no objection to giving soups,, animal broths rice or tapioca.

Because of the great tendency to anemia, the patient should be placed on a liberal diet as soon as the temperature is normal.

Much has been claimed for the alkaline treatment of rheumatism, that is shortens the course, and that it prevents the development of endocarditis by depressing the circulation. Though the alkalies may be useful adjuncts to treatment, no drugs give such marked beneficial results as the salicylates; indeed, we have come to look upon them as a specific for this disease. The best form is the sodium salicylate from the true oil of wintergreen itself, but aspirin or salicin may be tried. The hospital routine is to give twenty grains of sodium salicylate every hour until the patient is toxic, the total amount not to exceed 200 grains. If the patient is then not toxic and the disease has not yielded to treatment, he continues giving the salicylate, watching closely the effect of each dose, until in some cases as much as 300 grains have been taken. There is less likelihood of disturbance of the stomach if the salicylates are given well diluted, and in combination will sodium bicarbonate. It is gratifying indeed to see the remarkable transformation in the patient. Patients admitted in the afternoon with a temperature of 103 degrees and joints so painful that they scream with pain if the bed is touched, next morning have a normal temperature and move their joints freely, the pain, tenderness, and swelling having all disappeared. Often cases which have been treated outside the hospital with small doses of salicylates without benefit, have rapidly cleared up when the drug was pushed to its toxic limit. Seldom is the stomach disturbed. The toxic symptoms noted are ringing in the ears and deafness.

Local treatment of the joints is of secondary importance, though it is very comforting in the acute stage. Oil of wintergreen should be painted on the joints, and they should be wrapped in thick cotton wadding. Great relief is sometimes given by the application of hot stupes of lead and opium, or by the application of menthol and oil of wintergreen ointment.

After the acute symptoms have subsided the local applications are discontinued, and 15 to 20 grains of sodium salicylate is given three times a day. To ward off anemia, iron should be prescribed early. The patient should be kept in the recumbent posture for three weeks to avoid cardiac complications. This is especially difficult with the salicylate treatment, as the patient feels so well in two or three days that he often demands to be allowed up.—Cleveland Medical Journel.

